# A Novel Mutation in the *TECTA* Gene in a Chinese Family with Autosomal Dominant Nonsyndromic Hearing Loss

**DOI:** 10.1371/journal.pone.0089240

**Published:** 2014-02-21

**Authors:** Yu Su, Wen-Xue Tang, Xue Gao, Fei Yu, Zhi-Yao Dai, Jian-Dong Zhao, Yu Lu, Fei Ji, Sha-Sha Huang, Yong-Yi Yuan, Ming-Yu Han, Yue-Shuai Song, Yu-Hua Zhu, Dong-Yang Kang, Dong-Yi HAN, Pu Dai

**Affiliations:** 1 Department of Otorhinolaryngology, Head and Neck Surgery, PLA General Hospital, Beijing, P. R. China; 2 Department of Otorhinolaryngology, Hainan Branch of PLA General Hospital, Sanya, P. R. China; 3 Department of Otolaryngology, Emory University School of Medicine, Atlanta, Georgia; 4 Department of Otorhinolaryngology, the Second Artillery General Hospital, Beijing, P. R. China; 5 Department of Otorhinolaryngology, the First Affiliated Hospital of PLA General Hospital, Beijing, P. R. China; Universitat Pompeu Fabra, Spain

## Abstract

*TECTA*-related deafness can be inherited as autosomal-dominant nonsyndromic deafness (designated DFNA) or as the autosomal-recessive version. The α-tectorin protein, which is encoded by the *TECTA* gene, is one of the major components of the tectorial membrane in the inner ear. Using targeted DNA capture and massively parallel sequencing (MPS), we screened 42 genes known to be responsible for human deafness in a Chinese family (Family 3187) in which common deafness mutations had been ruled out as the cause, and identified a novel mutation, c.257–262CCTTTC>GCT (p. Ser86Cys; p. Pro88del) in exon 3 of the *TECTA* gene in the proband and his extended family. All affected individuals in this family had moderate down-sloping hearing loss across all frequencies. To our knowledge, this is the second *TECTA* mutation identified in Chinese population. This study demonstrates that targeted genomic capture, MPS, and barcode technology might broaden the availability of genetic testing for individuals with undiagnosed DFNA.

## Introduction

Hearing loss is one of the most common sensory disorders in humans. Genetic factors account for more than 50% of the cases with congenital or prelingual hearing loss, with autosomal-recessive (77%), autosomal-dominant (22%), and X-linked (1%) inheritance [1]. Single-gene defects are responsible for over half of these cases and most are thought to affect the cochlea [2]. A common clinical scenario is the identification of a child with partial hearing loss who then slowly progresses to deafness over a period of years. Identification of the responsible mutation in families with autosomal-dominant nonsyndromic hearing loss (ADNSHL) is difficult because mutations in 27 different genes have been identified as causing this common form of deafness (Hereditary Hearing Loss Homepage). Although certain gene mutations affect inner ear-specific proteins and can be linked to progressive hair cell degeneration, the mechanisms behind most common causes of progressive hearing loss in childhood are unknown [3].

Mutations in the *TECTA* gene at the DFNA8/12 (OMIM 601543) and DFNB21 (OMIM 603629) loci result in ADNSHL and the autosomal-recessive version, respectively [4]–[17] (Table1, 2). The *TECTA* gene encodes alpha (α)-tectorin, a major extracellular protein in the tectorial membrane (TM), a ribbon-like strip of extracellular matrix that lies over the stereocilia of the hair cells and is critical for the mechanical transmission and amplification of sound [18]. α-Tectorin is a large modular glycoprotein that contains several protein–protein interaction domains: the entactin domain (ENT), the large zonadhesin region (ZA) containing three full and two partial von Willebrand factor (vWF) type D repeats, an N-terminal entactin G1-like domain, a C-terminal zona pellucida (ZP) domain, and three trypsin inhibitor-like cysteine-rich domains [19]–[21].

**Table 1:** *TECTA* mutations described in DFNA8/12 families, including those identified in this study.

Exon	Mutation	Protein change	Domain	Time of onset	Progression	Frequencies	Ethnic origin	Reference
3	c.257–262CCTTTC>GCT	p. Ser 86Cysp. Pro88del	ENT	Postlingual	Stable	High	Chinese	This study
4	c.589G>A	p.Asp197Asn	ENT	Postlingual	Stable	Mid	American	[7]
5	c.632T>C	p.Phe211Ser	ENT	Postlingual	Stable	Mid	Spanish	[7]
6	c.950T>A	p.Val317Glu	ZA (none)	Postlingual	Unknown	High	Korean	[17]
6	c.1084A>T	p.Ser362Cys	ZA (VWFD1)	Postlingual	Unknown	Mid	American	[7]
6	c.1124delT	p.Val375Alafs*4	ZA (VWFD1)	Postlingual	Unknown	Mid	Spanish	[7]
7	c.1395T>G	p.Asn465Lys	ZA (VWFD1)	Postlingual	Progressive	Mid	Belgian	[7]
7	c.1685C>T	p.Thr562Met	ZA (none)	Postlingual	Unknown	Mid	American	[7]
9	c.2444C>T	p.Thr815Met	ZA (VWFD2)	Prelingual	Unknown	Mid	American	[7]
9	c.2657A>G	p.Asn886Ser	ZA (VWFD2)	Prelingual	Progressive	High	UK	[7]
10	c.3107G>A	p.Cys1036Tyr	ZA (TIL2)	Postlingual	Stable	Mid	Spanish	[7]
10	c.3169T>A	p.Cys1057Ser	ZA (none)	Postlingual	Progressive	High	Swedish	[31]
10	c.3293C>T	p.Ala1098Val	ZA (none)	Postlingual	Unknown	High	Spanish	[7]
10	c.3406G>C	p.Asp1136His	ZA (VWFD3)	Postlingual	Unknown	High	Spanish	[7]
11	c.3743C>T	p.Pro1248Leu	ZA (VWFD3)	Prelingual	Unknown	High	Spanish	[7]
13	c.4525T>G	p.Cys1509Gly	ZA (VWFD4)	Unknown	Progressive	High	Turkish	[22]
13	c.4549T>C	p.Cys1517Arg	ZA (VWFD4)	Postlingual	Progressive	High	Spanish	[7]
14	c.4856G>C	p.Cys1619Ser	ZA (VWFD4)	Postlingual	Progressive	High	French	[32]
16	c.5331G>A	p.Leu1777Leu	ZA (none)	Prelingual	Stable	Mid	Dutch	[5]
16	c.5372C>G	p.Pro1791Arg	ZA (none)	Prelingual	Unknown	Mid	American	[7]
	c.5383+2T>G		ZA (none)	Prelingual	Stable	Mid	Spanish	[7]
	c.5383+5del GTGA		ZA (none)	Prelingual	Progressive	High	UK	[7]
17	c.5458C>T	p.Leu1820Phe	ZP	Postlingual	Stable	Mid	Belgian	[18]
17	c.5471G>A	p.Gly1824Asp	ZP	Postlingual	Stable	Mid	Belgian	[18]
17	c.5509T>G	p.Cys1837Gly	ZP	Postlingual	Progressive	Mid	Spanish	[33]
17	c.5509T>G	p.Cys1837Gly	ZP	Postlingual	Progressive	Mid	Spanish	[7]
17	c.5509T>C	p.Cys1837Arg	ZP	Postlingual	Progressive	Mid	American	[34]
18	c.5597C>T	p.Thr1866Met	ZP	Postlingual	Stable	Mid	Korean	[17]
18	c.5597C>T	p.Thr1866Met	ZP	Postlingual	Progressive, Unknown	Mid	Spanish, American	[7]
18	c.5600A>G	p.His1867Arg	ZP	Postlingual	Progressive	Mid	Spanish	[7]
18	c.5609A>G	p.Tyrl870Cys	ZP	Prelingual	Stable	Mid	Austrian	[18]
18	c.5668C>T	p.Arg1890Cys	ZP	Prelingual	Stable	Mid	Dutch	[15]
18	c.5668C>T	p.Arg1890Cys	ZP	Prelingual	Stable	Mid	Spanish American	[7]
18	c.5692T>C	p.Cys1898Arg	ZP	Postlingual	Unknown	Mid	American	[7]
19	c.5839C>T	p.Arg1947Cys	ZP	Postlingual	Unknown	Mid	American	[7]
19	c.5945C>A	p.Ala1982Asp	ZP	Prelingual	Progressive	Mid	Chinese	[35]
20	c.6026T>C	p.Ile2009Thr	ZP	Postlingual	Stable	High	Spanish	[7]
20	c.6062G>A	p.Arg2021His	ZP	Prelingual	Stable	Mid	Japanese	[9]

**Table 2:** DFNB21 *TECTA* mutations and the associated phenotypes.

Exon	Mutation	Protein change	Domain	Time of onset	Progression	Frequencies	Ethnic origin	Reference
3	c.266delT	p.Leu89Argfs*34	ENT	Prelingual	Stable	Mid	Iranian	[11]
5	c.651dupC	p.Asn218Glnfs*31	ENT	Prelingual	Stable	All freq	Iranian	[14]
	c.2941+1G>A		ZA	Prelingual	ND	All freq		[36]
	9.6 Kb	del	ZA	Prelingual	Stable	Mid	Iranian	[11]
15	c.5211C>A	p.Tyr1737*	ZA	Prelingual	Stable	Mid	Iranian	[11]
20	c.6037delG	p.Glu2013Argfs*6	ZP	Prelingual	Stable	Mid	Pakistani	[14]
21	c.6203–6218del	p.Lys2068Argfs*38	ZP	Prelingual	Stable	All freq	Iranian	[4]

Mutations in *TECTA* (*DFNA8/12*) can cause either stable or progressive hearing loss, depending on the affected protein domain [22]. Mutations in the ZP domain are commonly associated with stable hearing loss, whereas mutations within the vWF domains tend to manifest as progressive hearing loss [15]. *TECTA* null mice are deaf because the TM is detached completely from the organ of Corti; consequently, vibrations of the basilar membrane associated with the traveling wave do not lead to the deflection of outer hair cell (OHC) or inner hair cell (IHC) stereocilia [23]. Mice carrying a mutation in the ZP domain have congenital hearing loss because they have a misshapen TM that stimulates OHCs normally, but under-stimulates IHCs [24].

Massively parallel sequencing (MPS), also known as next-generation sequencing (NGS), produces large amounts of genomic sequence information in an incomparably more rapid and less expensive manner than before [25]. Given its large capacity to survey the entire genome without bias, NGS is suited for discovering the causative mutations of hereditary hearing loss with genetic heterogeneity. Novel genes for non-syndromic [26], [27] and syndromic [28] hearing loss were identified recently using a targeted NGS approach. In this study, we first identified ADNSHL in Chinese Family 3187 caused by the c.257–262CCTTTC>GCT (p. Ser86Cys; p. Pro88del) mutation of *TECTA* using targeted capture of 42 deafness genes and MPS. To exclude the possibility that the mutation is a polymorphism in the Chinese population, we sequenced the mutation directly (c.257–262CCTTTC>GCT (p. Ser86Cys; p. Pro88del)) in 100 ethnicity-matched, unrelated controls. The c.257–262CCTTTC>GCT (p. Ser86Cys; p. Pro88del) variant co-segregated with the phenotype and was absent in the 100 ethnicity-matched controls. This is the first report of *TECTA* c.257–262CCTTTC>GCT (p. Ser86Cys; p. Pro88del) as a gene causing ADNSHL in Chinese families with hereditary deafness.

## Materials and Methods

### Subjects and Ethics Statement

We enrolled six cases of ADNSHL from Family 3187, a large ethnic Han Chinese family, through the Otolaryngology Department of the General Hospital of People’s Liberation Army, Beijing, China clinical evaluations, temporal bone imaging results, audiograms, and other relevant clinical information were collected for each member. The proband had no obvious syndromic symptoms, and *GJB2* mutations, *SLC26A4* hotspot mutations, and mtDNA1494 and 1555 mutations were excluded.

**Figure 1:**
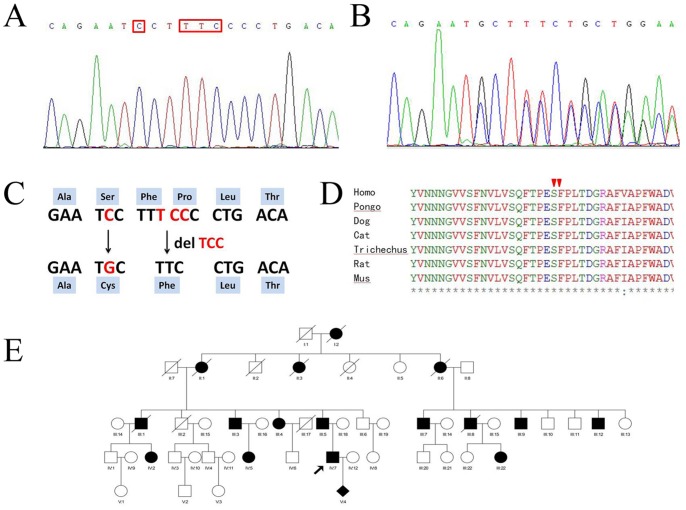
The c.257–262CCTTTC>GCT mutation detected in Family 3187. (**A**) Partial DNA sequences of the *TECTA* gene in a normal family member and (**B**) the c.257–262CCTTTC>GCT mutation (in family members IV:2, IV:7, III:4, III:5, III:3, IV:5). (**C**) The amino acid changes caused by the changes in the DNA sequence. (**D**) Alignment of the α-tectorin and homologous sequences in the N-terminal ENT-like interdomain. The arrow marks the position of the c.257–262CCTTTC>GCT mutation (p. Ser86Cys; p. Pro88del). The ENT-like domain is highly conserved in different species. (**E**) The pedigree of Family 3187 for the c.257–262CCTTTC>GCT mutation. The solid symbols indicate affected individuals.

Pure-tone audiometry was performed in a sound-controlled room at frequencies ranging from 250 to 8000 Hz, according to standard protocols. Audiograms were available for 6 of the 10 affected members (IV:2, IV:7, III:4, III:5, III:3, IV:5) and 6 unaffected individuals (III:6, III:18, IV:1, IV:3, IV:4, IV:6) (Fig. 1c). The audiology results were characterized in terms of hearing level and audiogram shape.

The study was approved by the Chinese PLA General Hospital Research Ethics Committee. After obtaining written informed consent, blood samples were obtained from 12 family members. Consent was attained from each subject or their guardians.

### Target Enrichment, Capture Libraries, MPS

The quality of the genomic DNA (gDNA) was examined by checking the optical density ratio (260/280 ratio) and performing gel electrophoresis. High-molecular-weight gDNA (∼5 µg) was fragmented ultrasonically with a Covaris E210 DNA shearing instrument (Covaris, Woburn, MA) to an average size of 200 base pairs (bp). The Covaris protocol was set as follows: 3 min total duration, duty cycle 10%, intensity 5, and 200 cycles per burst.

The exons and flanking 50 bp of 42 known human deafness genes were selected for capture and NGS sequencing with an Illumina Genome Analyzer IIX (Solexa). Hybridization probes (0.5∼1.6 kilobase pairs) for these genes were generated from either cDNA clones of the genes or by polymerase chain reaction (PCR) amplification of targeted gDNA regions. To ensure reliable capture of shorter exons, we specifically generated longer hybridization probes from gDNA for those exons that were shorter than 50 bp by including about 100 bp gDNA flanking the exons on both sides. All PCR products (10 ng of each) were cleaned using the QIAquick PCR Purification Kit (QIAGEN, Valencia, CA) before use. More details of capture probe validation and preparation can be found in a previous report [29].

Fragmented gDNA libraries for Illumina GAII sequencing were prepared with the NEBNext™ DNA Sample Prep Master Mix set (E6040, New England Biolabs, Ipswich, MA). End repair of DNA fragments, the addition of a 3′ adenine (A), adaptor ligation, and reaction clean-up were carried out following the manufacturer’s protocol. The libraries were cleaned and size-selected using the AMPure DNA Purification kit (Beckman Agencourt, Danvers, MA). The ligated product (20 ng) was amplified for 14 PCR cycles with Illumina PCR primers InPE1.0, InPE2.0, and indexing primers, following the manufacturer’s instructions.

For targeted enrichment of deafness genes, the Illumina library DNA was purified with a QIAquick MinElute column and eluted into 50 µL hybridization buffer (HB, Roche NimbleGen, Madison, WI). The barcoded Illumina gDNA libraries (5 µg) were incubated in 16 µL HB on the surface of hybridization glass slides on a hybridization station (BioMicro Systems, Salt Lake City, UT) at 42°C for 72 h. Nonspecific DNA fragments were removed after six washing steps in washing buffer (Roche NimbleGen, Madison, WI). The DNA bound to the probes was eluted by incubating it with NaOH (425 mL, 125 mM) for 10 min. The eluted solution was transferred to a 1.5 mL Eppendorf tube containing 500 µL neutralization buffer (QIAGEN PBI buffer). The neutralized DNA was desalted and concentrated on a QIAquick MinElute column and eluted into 30 µL EB buffer. To increase the yield, we amplified 5 µL eluted solution with 12 PCR cycles using Illumina PCR primers InpE1.0 and InpE2.0. The enrichment of targeted deafness genes was examined by quantitative PCR (qPCR), comparing the growth curves of captured and non-captured samples [30]. Twelve barcoded libraries of captured samples were pooled and paired-end Illumina sequencing was performed with the Illumina HiSeq system (Illumina, San Diego, CA). Details of the bioinformatic analysis methods were previously published [29]. Sequence read data of the patient in this article has been deposited into Sequence Read Archive (http://www.ncbi.nlm.nih.gov/sra webcite; accession number SRR1106702).

### Sanger Sequencing

After filtering against multiple databases, Sanger sequencing was used to determine if any of the potential novel mutations in known genes causing ADNSHL co-segregated with the disease phenotype in this family. Sequencing was performed using the ABI 3130 Avant capillary electrophoresis system.

### Prenatal Diagnosis

After confirming the genetic defect in Family 3187, we performed prenatal diagnosis for the proband’s wife in the 11^th^ week of pregnancy. After obtaining informed consent from the proband and his wife, we performed transabdominal chorion villus sampling under ultrasound guidance. The DNA from the chorion was extracted using a chorion DNA extraction kit (TianGen, Beijing, China). PCR amplification and sequencing of TECTA from the chorion was performed under the same conditions as those used for the blood samples.

## Results

### Mutation Analysis of the TECTA Gene

**Figure 2:**
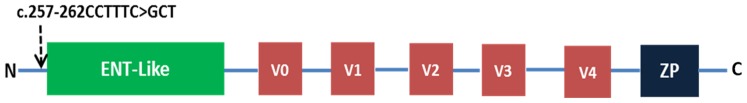
Domain structure of *TECTA*. The mutation lies within the N-terminal region, upstream from the ENT-like domain.

We identified a novel mutation (c.257–262CCTTTC>GCT [p. Ser86Cys; p. Pro88del]) in exon 3 (Fig. 1A) of the *TECTA* gene in Family 3187, and confirmed the two mutations at the same allele. The mutation in the coding region of *TECTA* results in two amino acid substitutions: serine to cysteine at position 86 and a deletion of phenylalanine at position 87. The affected members in this family (Fig. 1C) were heterozygous for this mutation, while the mutation was not observed in family members with normal hearing or in 100 unrelated subjects. This mutation was located in the N-terminal region, upstream from the ENT-like domain (Fig. 2), which is highly conserved across species (Fig. 1B). The inheritance pattern of hearing loss in this family is autosomal dominant. This evidence supports the inference that c.257–262CCTTTC>GCT is a pathogenic mutation rather than a rare polymorphism.

The prenatal diagnosis results revealed that the fetus (P0818374) carried the mutation *TECTA* (c.257–262CCTTTC>GCT [p. Ser86Cys; p. Pro88del]) and was predicted to inherit his father’s hearing (3187–1).

### Audiological Features

**Figure 3:**
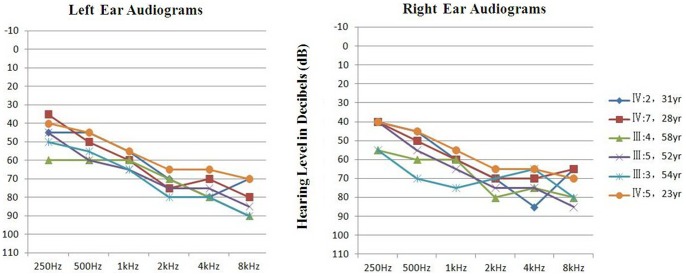
Pure-tone audiograms for the left and right ears of six affected individuals (IV:2, IV:7, III:4, III:5, III:3, IV:5). The numbers to the right are the individuals’ ages.

In Family 3187, the affected members developed progressive down-sloping bilateral hearing loss beginning in their teens. All six subjects had bilaterally symmetrical hearing loss with average thresholds of 0.5, 1, 2, and 4 kHz (4FA) of 61∼70 dB hearing loss in both ears, demonstrating severe hearing impairment according to the WHO 1997 criteria (Table 3). None of the affected subjects had a history or evidence of any other cause of hearing loss (Fig. 3).

**Table 3:** Grades of hearing impairment (WHO1997).

Grade of impairment	Corresponding audiometricISO value
0– None	25 dB or better
1– Slight	26–40 dB
2– Moderate	41–60 dB
3– Severe	61–80 dB
4– Profound, including deafness	81 dB or greater

Grades 2 to 4 are classified as disabling hearing impairment. The audiometric ISO values are averages of values at 500, 1000, 2000, 4000 Hz.

## Discussion

According to the second nationwide survey of the disabled Chinese population performed in 2006, there were 27.80 million people with hearing and speech disabilities in China; of these, 20.04 million have simple hearing disabilities, with genetic factors accounting for about 50% of the Chinese deaf population of Han ethnicity [37]. Of these cases, almost 80% show autosomal-recessive inheritance. In China, routine clinical diagnostic tests for deafness have consisted of the GJB2 [38], [39], SLC26A4 [40], and mitochondrial 12S rRNA genes [41], [42]. So far, there is no clinical genetic diagnosis for ADNSHL in China.

ADNSHL is a common and often progressive sensory deficit. There are currently 64 mapped loci for this condition, but only 25 causative genes have been identified. In general, the ADNSHL phenotypes are postlingual hearing loss mostly affecting the high frequencies, although some hereditary hearing loss involves mainly low or intermediate frequencies. The latter is seen in cases with mutations at the DFNA8/12, DFNA13, DFNA21, DFNA31, DFNA44, and DFNA49 loci. In the majority of these traits, some progression is observed. DFNA8/12 hearing loss is caused by mutations in the *TECTA* gene, which encodes the TM protein α-tectorin. Depending on the exons carrying the mutation and thus the protein domains affected, *TECTA* mutations cause either mid- or high-frequency hearing loss. Michael et al. (2011) completed unbiased screening for *TECTA* mutations in a Spanish cohort of 372 probands from ADNSHL families and identified 20 novel mutations in 23 *TECTA* mutations, more than doubling the number of reported *TECTA* ADNSHL mutations from 13 to 33. They postulated that DFNA8/12 hearing loss is one of the most common forms of ADNSHL in the Spanish population and suggested that its global prevalence is about 4% of all ADNSHL [7].

We discovered a novel mutation in exon 3 of the *TECTA* gene in Family 3187: c.257–262CCTTTC>GCT (p. Ser86Cys; p. Pro88del). This was implicated as a pathogenic mutation causing hearing loss. In families with *TECTA* ADNSHL, all reported changes are missense mutations that cause a dominant-negative effect. The mutation c.257C>G located within exon 3 in the N-terminal region of *TECTA*, upstream from the ENT-like domain, resulted in a single amino acid substitution: serine to cysteine. The mutation c.260delTTC results in the deletion of phenylalanine at position 87. Two mutations in this domain have been reported: c.266delT and c.248C>T (p.T83M). Meyer et al. [11] identified three novel homozygous *TECTA* gene mutations in Iranian families, including one frameshift mutation (266delT) in exon 3 that creates a stop codon at position 122 of the protein (122X). Plantinga et al. [43] detected two changes (p.R1890C and p.T83M) in α-tectorin in a family with autosomal-dominant middle-frequency hearing impairment. The mutation c.248C>T (p.T83M) was not in a specific domain and seemed to be a relatively mild amino acid substitution according to the Dayhoff table. However, because this change was not present in controls, it might have affected the phenotype of these patients or even acted synergistically with the p.R1890C mutation [15].

Mutations of the ENT-like domain may cause sensorineural hearing loss. Exon 3 has 287 nucleotides and encodes 96 amino acids (66–162). Together with type IV collagen, laminin, and heparin sulfate proteoglycans, entactin is a major component of basement membranes where it facilitates cell adhesion by binding to calcium ions [44]. Entactin is predicted to interact with laminin and type IV collagen by acting as a bridge and inducing their deposition in the extracellular matrix [45], [46]. Therefore, it is possible that the entactin domain of *TECTA* facilitates the assembly and modeling of the extracellular matrix of the TM and that the identified mutations could drastically affect its role during the mechanotransduction of sound.

The *TECTA* gene encodes α-tectorin, which plays an important role in the structure and function of the TM. In mice, targeted deletion of the *TECTA* gene results in complete detachment of the TM from the organ of Corti [23]. In humans, nearly all recessive DFNB21 mutations in *TECTA* result in premature stop codons that may result in either truncated α-tectorin protein products or nonsense-mediated degradation of the *TECTA* mRNA, and are considered loss-of-function mutations [11], [14], [36]. The DFNA8/12 mutations in *TECTA*, which cause dominant hearing loss, all substitute highly conserved amino acids. The various missense mutations in *TECTA* that cause DFNA8/12 can be subdivided into classes with a clear genotype/phenotype correlation.

The phenotype associated with dominant *TECTA* mutations usually depends on the affected α-tectorin domain. Structural variation in the ZP and ZA domains is common, and most of it likely causes functional problems in α-tectorin. Mutations that affect the vWFD2-D3 inter-repeat connector or vWFD4 repeat from the ZA domain are associated with progressive hearing loss at high frequencies [7]. Conversely, mutations in regions other than the ZA, entactin, or ZP domains, with few exceptions, are associated with mid-frequency hearing loss and result in a flat-to-shallow U-shaped audiometric profile [7], [15]. This type of correlation does not exist in familial ADNSHL.

All of our affected subjects had audiogram configurations showing mild ski-slope loss, with greater hearing loss at high frequencies than at low frequencies. The average hearing thresholds at low frequencies of 0.25to 1 kHz were in the range of 40–60 dB hearing loss, whereas those at frequencies higher than 2 kHz were in the range of 60–80 dB hearing loss. The tympanograms were type A, indicating normal function of the middle ear and Eustachian tube. Recruitment was revealed in the subjects by the lower dynamic range between hearing and stapedial acoustic reflex thresholds, which indicated that the lesions were in cochlea. The patients had absent distortion-product otoacoustic emissions at all frequencies, while auditory brainstem responses were present with normal inter-peak latencies. These results demonstrate that the nonlinear compression function of the cochlea was impaired and suggest that the lesion was near the outer hair cells. The *TECTA* gene encodes the α-tectorin protein in the striated sheet matrix, whereas the TM is an acellular gelatinous structure that contacts the hair cells in the organ of Corti; therefore, mutation of the *TECTA* gene leads to hearing loss.

Worldwide, of the 33 reported DFNA8/12 mutations, only 4 (p.Cys1036Tyr, p.Cys1837Gly, p.Thr1866Met, and p.Arg1890Cys) have been observed in multiple unrelated families. It is difficult to research ADNSHL in depth because of the cost and time involved. In the past few years, NGS technologies have made great strides in both basic and clinical research, providing deeper insights into the complex genomic landscapes of many diseases. Due to the high genetic heterogeneity of hearing loss, targeted DNA capture and MPS appear to be ideal tools for investigating this disease.

The English in this document has been checked by at least two professional editors, both native speakers of English. For a certificate, please see:

http://www.textcheck.com/certificate/sTwy8P.
